# 3D Printing of Porous Scaffolds with Controlled Porosity and Pore Size Values

**DOI:** 10.3390/ma11091532

**Published:** 2018-08-25

**Authors:** Irene Buj-Corral, Ali Bagheri, Oriol Petit-Rojo

**Affiliations:** Department of Mechanical Engineering, Universitat Politècnica de Catalunya, Av. Diagonal, 647, 08028 Barcelona, Spain; ali.bagheri@upc.edu (A.B.); oriol.petit@hotmail.com (O.P.-R.)

**Keywords:** fused deposition modeling, 3D printing, scaffolds, porosity, pore size, multiobjective optimization

## Abstract

3D printed scaffolds can be used, for example, in medical applications for simulating body tissues or for manufacturing prostheses. However, it is difficult to print porous structures of specific porosity and pore size values with fused deposition modelling (FDM) technology. The present paper provides a methodology to design porous structures to be printed. First, a model is defined with some theoretical parallel planes, which are bounded within a geometrical figure, for example a disk. Each plane has randomly distributed points on it. Then, the points are joined with lines. Finally, the lines are given a certain volume and the structure is obtained. The porosity of the structure depends on three geometrical variables: the distance between parallel layers, the number of columns on each layer and the radius of the columns. In order to obtain mathematical models to relate the variables with three responses, the porosity, the mean of pore diameter and the variance of pore diameter of the structures, design of experiments with three-level factorial analysis was used. Finally, multiobjective optimization was carried out by means of the desirability function method. In order to favour fixation of the structures by osseointegration, porosity range between 0.5 and 0.75, mean of pore size between 0.1 and 0.3 mm, and variance of pore size between 0.000 and 0.010 mm^2^ were selected. Results showed that the optimal solution consists of a structure with a height between layers of 0.72 mm, 3.65 points per mm^2^ and a radius of 0.15 mm. It was observed that, given fixed height and radius values, the three responses decrease with the number of points per surface unit. The increase of the radius of the columns implies the decrease of the porosity and of the mean of pore size. The decrease of the height between layers leads to a sharper decrease of both the porosity and the mean of pore size. In order to compare calculated and experimental values, scaffolds were printed in polylactic acid (PLA) with FDM technology. Porosity and pore size were measured with X-ray tomography. Average value of measured porosity was 0.594, while calculated porosity was 0.537. Average value of measured mean of pore size was 0.372 mm, while calculated value was 0.434 mm. Average value of variance of pore size was 0.048 mm^2^, higher than the calculated one of 0.008 mm^2^. In addition, both round and elongated pores were observed in the printed structures. The current methodology allows designing structures with different requirements for porosity and pore size. In addition, it can be applied to other responses. It will be very useful in medical applications such as the simulation of body tissues or the manufacture of prostheses.

## 1. Introduction

Many new applications have arisen as a result of recent advances in 3D printing techniques. For example, printed parts are used for manufacturing space instrumentation, for both prototypes and flying parts [1], for manufacturing cost-effective parts in the sports industry or for developing new protective structures for vehicles in the automotive industry [2]. 3D printing has many different medical applications, such as bioprinting tissues and organs, building vascularized organs, the manufacture of customized implants, prostheses and models for surgical preparation, among others [3]. Thus, 3D printed scaffolds can be employed as templates for initial cell attachment and tissue formation, for example in bone tissue engineering [4]. They can also be used for fixing prostheses by means of osseointegration. Scaffolds could be printed in different materials such as titanium, degradable polymers and degradable ceramics [5,6]. Specifically, in FDM or fused deposition modelling technique a material is melted through an extrusion head and deposited layer by layer [7]. Main advantages of FDM technology are its easiness of use and the fact that it allows printing a wide range of materials, as long as they can be extruded, for example plastic materials such as acrylonitrile butadiene styrene (ABS) or polylactic acid (PLA). It is also more cost-effective than other additive manufacturing techniques, and its lead times are short. However, it also has disadvantages. It does not provide high dimensional precision, layer steps are usually observed on the part’s surface, causing the surfaces not to be smooth. Besides, the use of and the use of scaffolds with biocompatible materials is difficult, because the technology is limited to materials whose viscosity is sufficiently low that they can be extruded, but high enough so that their shape is maintained after extrusion [8,9,10].

Regarding the design of 3D printed porous scaffolds that simulate tissues, some properties to keep in mind are: surface area and interconnectivity, which are related to cell growth; permeability, which governs nutrient transport; and mechanical strength, which assures support and protection, among other properties.

One possibility to achieve required porosity is to use hierarchical scaffold design, creating libraries of unit cells that can be joined to obtain scaffold structures. Hollister observed that increasing the material volume of a certain structure increased elastic modulus and decreased permeability [11]. In the same line, Egan et al. defined four different types of scaffold structures, taking into account either beam-based unit cells or truss-based unit cells. In addition, each structure could be created by means of either continuous or hierarchical patterning. They found that, given a certain porosity value, truss-based scaffolds have higher surface areas and lower elastic moduli than beam-based ones [12]. On the other hand, Arabnejad et al. presented a visualization method that allows understanding the relationship among cell topology, pore size and porosity in stretch-dominated structures, such as tetrahedron and octet trusses [13].

Another possibility to create structures with required porosity is use of topology optimization, which consists of distributing material in regions having low and high material density respectively. This is achieved by periodically repeating a unit cell, which is composed of areas with and without material [14,15]. When applying topological optimization methods, several authors have carried out multiobjective optimization of different properties in scaffolds. Lin et al. used an objective function that assigned different weights to the two responses considered: porosity and stiffness. The function was then maximized or minimized by numerical methods [16]. In a similar way, Guest and Prévost [17], as well as Hollister and Lin [18] used topology optimization to maximize stiffness and fluid permeability. Kang et al. used a homogenization-based topology optimization method to achieve the required bulk modulus and isotropic diffusivity for a certain porosity value [19]. Other properties of the structures, such as thermal conductivity [20], have been addressed. On the other hand, although the increase in surface area of pores helps tissue growth, this growth is facilitated if concave surfaces are used. In this direction, Egan et al. modelled scaffolds with curvature. They first fixed required porosity to assure a certain permeability value, which is desirable for nutrient transport, and then addressed the problem of mechanical strength of structures [21].

Different authors have tested the properties of FDM printed porous scaffolds. Regarding mechanical properties, Habib et al. used finite element analysis and compressive strength tests [22]. Wang et al. printed scaffolds for vascularized bone tissues in different materials, in order to test both their biomimetics and their strength [23]. Aw et al. tested the effect of printing parameters on tensile strength of conductive acrylonitrile butadiene styrene/zinc oxide (CABS/ZnO) composites [24]. Helguero et al. modelled artificial bones and printed them in acrylonitrile butadiene styrene (ABS). They tested both anisotropy and compressive strength of the scaffolds [25]. As for porosity, Gregor et al. printed PLA scaffolds and measured their porosity by means of X-ray microtomography [26]. Regarding surface finish, Townsend et al. listed most usual methods for measuring roughness profiles (contact stylus), and surface topography (confocal microscopy, focus variation microscopy, coherence scanning interferometry, chromatic confocal microscopy, conoscopic holography, atomic force microscopy (AFM), and elastomeric sensors [27]). Krolczyk et al. compared the roughness obtained in turning processes with that obtained in FDM processes [28]. They observed that the machined surface had an anisotropic and periodic structure, while the printed surface had an undirected structure. With the manufacturing conditions employed, the FDM process showed higher roughness values than the turning process.

In the present paper, a model was developed to define the pore size and the porosity of porous structures. Unlike other methods that are based on truss structures, the present model allows obtaining irregular porous structures from random location of columns in the space, which leave voids among them. Specifically, the structure was modelled with parallel planes joined by columns, with a certain number of columns on each plane. The model was applied to a disk shape. Three variables were defined: the distance between parallel planes, the number of base points for columns on each plane, and the radius of each column. Next, dimensional analysis was used to reduce the number of process variables to 2. Then, the requirements were defined for a specific application case: the use of a porous structure in external layers of hemispherical hip prostheses. Subsequently, the design of experiments, with three-level factorial analysis, was used to obtain mathematical models for porosity, mean of pore size and variance of pore size as a function of dimensionless variables. They allowed multiobjective optimization in order to determine the optimal values for the process parameters.

In order to compare experimental results to computationally calculated ones, samples were printed with FDM technology, and total porosity, as well as pore size, was measured. X-ray tomography was used to determine the total porosity of the printed structures by means of computation of plastic volume and comparison with total volume of the printed shape.

The present study will help designing and manufacturing porous structures with specific requirements regarding the porosity and pore size that favour osseointegration. The same methodology can be used, however, to achieve other requirements of porous structures, such as mechanical strength and/or nutrient transport.

## 2. Materials and Methods

### 2.1. Model for the Porous Structure

#### 2.1.1. Model Definition

**Figure 1:**
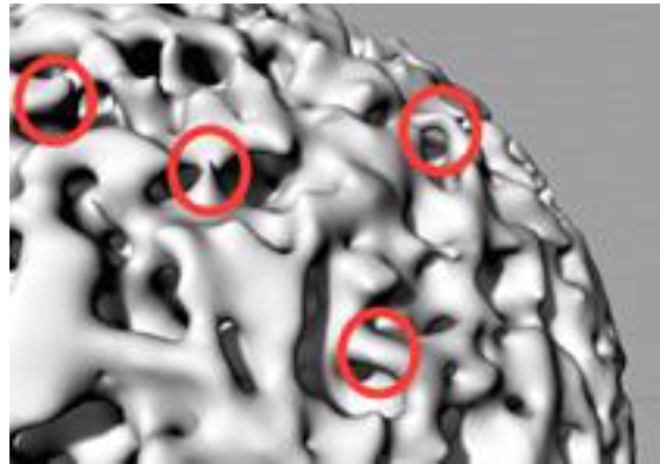
Trabecular structure with thin walls.

The printing process of a random porous structure, such as a trabecular one, presents some difficulties: the walls in certain parts of the structure are too thin (Figure 1), there are some areas with burrs, some parts of the structure have a high inclination angle that leads to the use of printing structure supports, etc.

**Figure 2:**
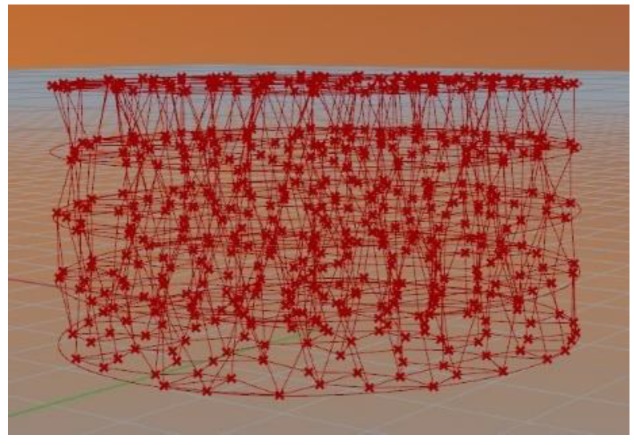
Structure obtained after applying the model to a disk shape.

Such difficulties can be attributed to the fact that, when designing the structure, a completely random distribution of points in space is used. Since distribution is random, connections between different points in space can have any orientation. In the present paper, a proposed solution for modelling porous structures is presented. It involves defining some theoretical parallel planes, each one of them with several theoretical points that are randomly distributed. Once the points have been created on the surfaces, it is necessary to connect them by means of theoretical lines. For doing this, each point is connected to the three nearest points of the same plane or of the plane that is immediately below. Thus, almost vertical lines will be created that will have a correct inclination angle for printing. Figure 2 shows a scheme of the model applied to a disk shape.

**Figure 3:**
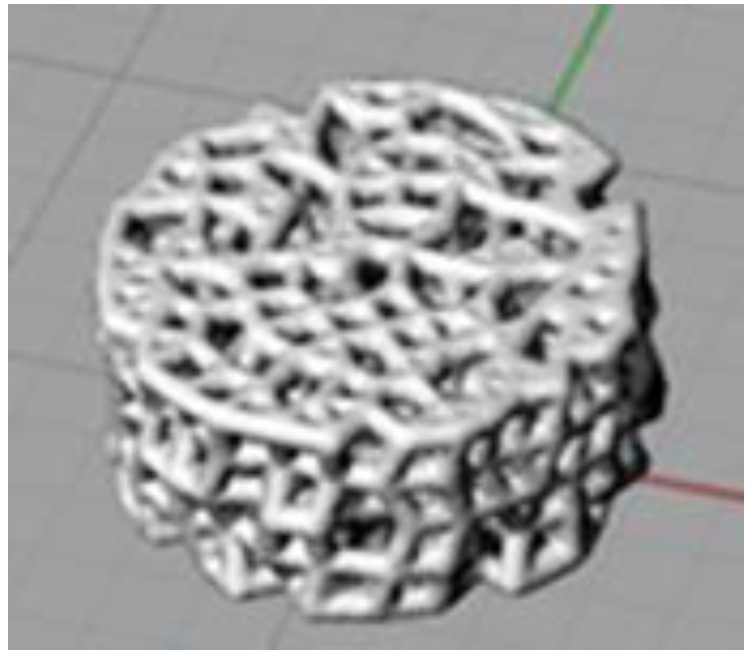
Structure obtained after applying the marching cubes algorithm to a disk shape.

The marching cubes algorithm allows lines and points to be given volume [29]. It is used in the present paper to create columns of the structure from the theoretical lines. The structure obtained after applying the model and the marching cubes algorithm to a disk shape is shown in Figure 3.

**Figure 4:**
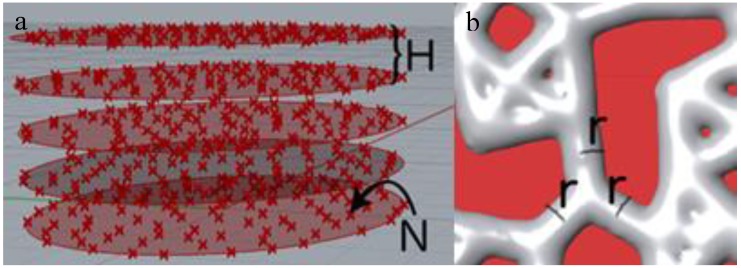
Parameters that define the porous structures: (**a**) parameters H and N, and (**b**) parameter r.

Thus, there are three parameters to be controlled: distance between parallel planes H (mm), number of points per surface unit on a certain plane N (points/mm^2^) (Figure 4a), and radius r of columns that are created around the model lines (mm) (Figure 4b).

With the aim of determining optimum values for parameters, it is necessary to find the relationship between the three model parameters and the responses of porosity and pore size. To do so, a design of experiments was used to obtain mathematical models and perform multiobjective optimization. Before optimization, a reduction of the variables was applied so that each response considered depends on two dimensionless variables.

#### 2.1.2. Reduction of Variables

Dimensional analysis allows reducing number of variables in a certain system [30]. If the problem depends on n dimensional variables, dimensional analysis reduces the problem to k dimensionless variables, where reduction is n − k = 1, 2, 3, or 4 depending on the complexity of the system. One of the most used techniques for reducing the number of variables is the Buckingham Pi theorem. This allows obtaining dimensionless parameters that are a product of powers named П_1_, П_2_, П_3_, etc. [31].

First part of the Pi theorem explains how to find reduction j Equation (1):

(1) j=n−k

where n is the number of variables of the problem and k is the number of dependent variables. From n variables, k variables are chosen as basic and the rest are scale variables.

The second part of the Pi theorem shows that each dimensionless parameter is defined as the product of the rest of variables with a proper exponent different than zero. In this way, all dimensionless groups are independent. Each dimensionless parameter is related to the other ones by means of a function Equation (2):

(2) $$\Pi_{1}=g\left(\Pi_{2},\Pi_{3},\Pi_{4},\;\ldots \right)$$

where Π_1_ is a dimensionless variable, and Π_2_, Π_3_, Π_4_ are other variables.

In the present paper, three responses were analysed separately: porosity p (dimensionless), mean of pore size M (length) and variance of pore size V (length^2^), with process variables H (length), N (length-2) and r (length). Variable r was chosen as the scale variable.

For porosity, dimensionless parameters are described in Equations (3)–(5):

(3) $$\Pi_{1}={\left(H\left[{\mathrm{long}}^{1}\right]\right)}^{1}\cdot {\left(r\left[{\mathrm{long}}^{1}\right]\right)}^{-1}=\frac{H}{r}\left[\mathrm{dimensionless}\right]$$

(4) $$\Pi_{2}={\left(N\left[{\mathrm{long}}^{-2}\right]\right)}^{1}\cdot {\left(r\left[{\mathrm{long}}^{1}\right]\right)}^{2}=N\cdot r^{2}\left[\mathrm{dimensionless}\right]$$

(5) $$\Pi_{3}=p\left[\mathrm{dimensionless}\right]$$

and the function to be found is presented in Equation (6):

(6) $$\Pi_{3}=g\left(\Pi_{1},\Pi_{2}\right)\to p=g\left(\frac{H}{r},N\cdot r^{2}\right)$$

Similar expressions were found for average value (M) and variance (V) of the probability density function and of the pore diameters Equations (7) and (8):

(7) $$\frac{M}{r}=j\left(\frac{H}{r},N\cdot r^{2}\right)$$

(8) $$\frac{V}{r^{2}}=k\left(\frac{H}{r},N\cdot r^{2}\right)$$

Thus, it was only necessary to perform experiments with two dimensionless factors, H/r and N·r^2^.

#### 2.1.3. Requirements of the Structure

The kind of porous structure that is defined in the present paper can be used, for example, to replace or simulate bone trabecular tissue. At the macroscopic level, bones are formed by cortical and trabecular compartments. Bone tissues have several functions, for example, support, protection, mineral storage, and nutrient transport [32]. If a printed structure is to be fixed by means of osseointegration, a certain degree of porosity is required. Two main factors governing the porous external surface are the pore size and the porosity of the structure. According to Karageorgiou [33] and Baino [34], optimum values of pore size were considered to range between 100 and 500 µm for a trabecular structure. The mean porosity value of scaffolds is recommended to be between 50 and 75% in volume. Although a certain variability of pore size, with small pores that improve cell attachment and large pores that favour nutrient transport could be desirable in some cases [35], in the present work only osseointegration was considered. For this reason, the variance of the pore size should take the lowest possible value, in order to assure that the maximum number of pores would lie within the interval. High connectivity between pores in the structures is also required, as porosity has to be accessible; an interconnecting channel structure is needed to allow tissue to grow on it.

### 2.2. Design of Experiments

In order to determine the required values for the two model variables considered and to optimize porosity, design of experiments was performed with simulated experiments. A full factorial design was employed with two dimensionless factors: H/r, the distance between surfaces where points are generated over a radius of generated volumes, and N·r^2^, the number of points per area unit. Three levels were defined for each factor and three replicates were performed for each experiment (since variability among replicates is high). In all, 3 × 3^2^ = 27 runs were carried out. The radius of columns remained constant in all experiments.

Levels for variables were selected according to previous tests. A summary of levels is presented in Table 1.

Responses considered were porosity, mean of pore diameters (mm), and variance of pore diameters (mm^2^), in order to consider both the mean and dispersion of the probability function of porosity.

In order to calculate theoretical porosity and distribution of pore diameters, a program created by Dupuy et al. was used [36]. It is a MATLAB (MathWorks, Natick, MA, USA) script that, from a binary 3D image, returns the function of cumulated porosity vs. pore diameter. In the present study, a distribution of points in space was created with the program Rhinoceros 3D with the Grasshopper plug-in (Robert McNeel & Associates, Seattle, WA, USA).

### 2.3. Multiobjective Optimization

Once the mathematical models had been obtained, it was necessary to choose values for the parameters (N, H, r) that provided the required values for porosity, average pore size, and variance, taking into account the requirements of the responses.

The desirability function method was employed for the multiobjective optimization of responses [37]. It is based on defining desirability functions $d_{i}\left(x_{i}\right)$ for each response. The range for the desirability function is [0, 1]. Value 0 is assigned to a situation that is undesirable and value 1 is assigned to a completely desirable situation.

The objective function to maximize is the geometric average of the group of desirability functions. Taking into account different weights k_i_ for each objective Equation (9):

(9) $$D=\sqrt[{\sum_{i=1}^{n}k_{i}}]{\prod_{i=1}^{n}{\left(d_{i}\left(x_{i}\right)\right)}^{k_{i}}}$$

where n is number of responses to be optimized at the same time, and $k_{i}$ defines the importance of each desirability function inside the objective function.

There are three kinds of desirability functions depending on the optimization goal: maximizing, minimizing, and target value desirability functions. As an example, in Equation (10) the maximization function is defined:

(10) $$d_{i,\max}\left(x_{i}\right)=\{\begin{array}{cc} 0 & x_{i}\leq L\\ {\left(\frac{x_{i}-L}{U-L}\right)}^{b} & L<x_{i}<U\\ 1 & U\leq x_{i} \end{array}$$

where L is a lower limit value from which the function becomes undesirable (di = 0) and U is an upper limit value from which the function is considered to be maximized (di = 1). Exponent b is a parameter that models the shape of the central part of the desirability function. It can take any value within [0, ∞]. If b takes values [0, <1], the central part of the function will be concave, and a small increment of the response from the unacceptable point leads to a great increment of d_i_. If b takes a value of 1, the central part of the function will be a straight line with constant slope. If b takes values [>1, ∞], the central part of the curve will be convex, and, until a value that is very similar to the optimal one is obtained, the d_i_ value will be almost zero.

Table 2 shows the selected values of parameters for optimization.

For porosity and mean of pore size, lower (L) and upper (U) values were selected, defining an interval for each response. The average value of the interval was selected as target value (T).

For variance of pore size, lower value (L) was 0, since variance is to be minimized so that the maximum number of pores is contained within the required interval for the pore size. The upper value (U) was set at 0.01. It is supposed that pore size follows a normal distribution, in which 95.4% of the population is included within the μ±2·σ interval. By equalling the range of pore diameters [0.100, 0.500] with the a.m. interval Equation (11), the maximal standard deviation σ_max_ = 0.100 mm is found, according to Equation (12). Thus, the maximal variance Variance_max_ is 0.010 mm^2^ according to Equation (13):

(11) $$\left[0.300-2\cdot \sigma_{\max},\;0.300+2\cdot \sigma_{\max}\right]\;\mathrm{mm}=\left[0.100,\;0.500\right]\;\mathrm{mm}$$

(12) $$\frac{0.300-0.100}{2}=0.100\;\mathrm{mm}=\sigma_{\max}$$

(13) $${\mathrm{Variance}}_{\max}={\sigma_{\max}}^{2}=0.010\;{\mathrm{mm}}^{2}$$

A MATLAB program was implemented in a discrete optimization of the objective function by means of sweeping. The program generates $m_{i}$ equispaced values within an interval for each variable (H, N, and r). Then, it checks if each point (combination of values for the different variables), meets the conditions defined in Table 1. Such conditions correspond to the space that is considered in the regression.

Table 3 corresponds to the intervals for variables and the *m*-values used for each variable. The value for m was selected so that the distance between contiguous values is lower than or equal to 0.005.

Different solutions to be found will depend on the importance ($k_{i}$) of each response. Specifically, four different cases were analyzed:

- The first case involves giving the same importance to all responses.
- The second case consists in giving higher importance to porosity response over the rest of the responses ($k_{porosity}=5$).
- The third case gives a higher importance to mean ($k_{mean}=5$).
- The fourth case consists in giving higher importance to variance ($k_{variance}=5$)

### 2.4. Experimental Tests

Printing experiments were performed in order to determine the experimental porosity of the samples. The techniques of fused filament fabrication (FFF) or fused deposition modelling (FDM) were selected. Cura software was used for generating the g-code for printing.

Disk-shaped samples of 6 mm diameter and 3 mm height were designed. Selected parameters for the structure were H = 0.72 mm, r = 0.15 mm, and N = 3.65 points/mm^2^ (according to results of multiobjective optimization in Section 3.2).

Three specimens were printed in a dual-extruder Sigma printer from BCN3D. Due to the difficulty to print the required columns of diameter 0.3 mm, the geometry of the disks was rescaled by a scaling factor of five before printing (30 mm diameter and 15 mm height). The printing speed of the head was set to 37 mm/s. The nozzle diameter was 0.2 mm, the layer height was 0.1 mm, and the infill was 40%. The shell thickness was 0.4 mm.

The geometry of the samples was measured by means of X-ray tomography with Zeiss Metrotom 800 equipment. From the geometry, it was possible to calculate the percentage of air (porosity) and the percentage of plastic in the structure with VG Studio Max software. From the 3D geometry, a cross-section was obtained at half the height of each specimen, where the width of pores was measured.

## 3. Results

In the present section results about the design of the experiments, multiobjective optimization, and experimental tests are presented.

### 3.1. Design of Experiments

Table 4 shows the results for the 27 different runs performed. The levels for the two factors considered are presented in codified units. Results for porosity, the mean of the pore size, and the variance of the pore size were simulated with a MATLAB program. Since the results of experiments come from simulation, the order of the runs is indifferent, and a standard order was used for performing the experiments.

Data were analysed with Minitab 17 (Minitab, State College, PA, USA). Multiple linear regressions were used to examine the mathematical models for the porosity, the mean of pore size, and the variance of the pore size as a function of the two dimensionless factors considered. For all models, hypotheses were formulated for the linearity, constant variance, normality, and independence of errors. All terms having *p*-values higher than 5% were not included in the model.

Equation (14) corresponds to porosity in real units:

(14) $$\mathrm{Porosity}=0.614+0.051{\left(\frac{H}{r}\right)}_{\mathrm{real}}-3.948{\left(N\cdot r^{2}\right)}_{\mathrm{real}}$$

The R-adj coefficient is 95.89%.

The function for the mean pore size in real units is shown in Equation (15):

(15) $$\mathrm{Mean}=0.929+0.024{\left(\frac{H}{r}\right)}_{\mathrm{real}}-11.336{\left(N\cdot r^{2}\right)}_{\mathrm{real}}+47.222{\left(N\cdot r^{2}\right)}_{\mathrm{real}}^{2}$$

The R-adj value is 96.29%.

Since variance does not follow a normal distribution pattern, the natural logarithm of variance was analysed, which shows a normal distribution. Equation (16) corresponds to real units for l n variance, while Equation (17) refers to variance:

(16) $$\ln\left(\mathrm{Variance}\right)=-3.552-16.061{\left(N\cdot r^{2}\right)}_{\mathrm{real}}$$

(17) $$\mathrm{Variance}=e^{-3.552-16.061{\left(N\cdot r^{2}\right)}_{\mathrm{real}}}$$

The R-adj coefficient is 44.14%.

Porosity depends on both H/R and N·r^2^, the mean pore size depends on the same variables and on (N·r^2^)^2^, while variance depends only on N·r^2^.

### 3.2. Multiobjective Optimization

Table 5 shows a comparison of all solutions found.

It was observed that radius r = 0.15 mm, which is the minimum value considered, is to be selected for all solutions. The recommended height found for all solutions is H = 0.72 mm. This value does not correspond to any limit for variable H. Finally, N varies depending on the solution considered.

Since porosity should be 0.625, with mean pore size 0.300 mm and minimal variance, it is not possible to achieve all objectives at the same time. As a result, depending on the importance given to any response, different N values are found.

**Figure 5:**
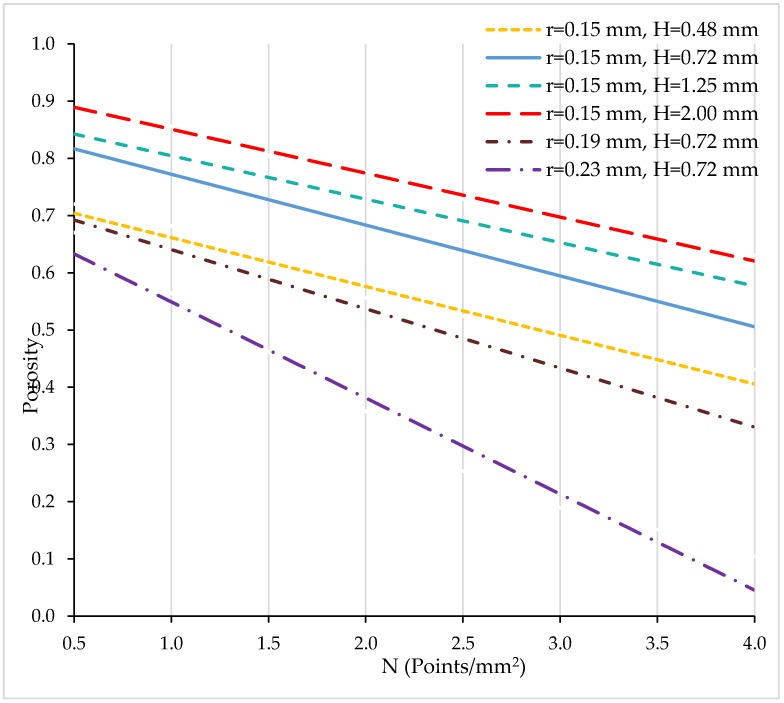
Porosity vs. N (points/mm^2^).

In Figure 5, curves for porosity are presented vs. N for different values of H and r. As a starting point, the solution found in the multiobjective optimization for equivalent importance of all responses was taken into account. It corresponds to the blue continuous line (H = 0.72 mm, r = 0.15 mm). From this solution, either height H or radius r were varied.

Porosity decreases linearly with number of points per surface unit. A broad range of porosity values can be achieved between 0.5 and 0.9 for the different combinations of H and r. For radius r = 0.15 mm, the higher the distance between parallel layers H, the higher the porosity (continuous and dashed lines). For radius r = 0.19 mm (dashed-dotted line in brown colour), the line shows a similar slope than for radius r = 0.15 mm. However, for higher radius r = 0.23, a steeper slope is observed and porosity decreases more sharply (dashed-dotted line in purple colour).

**Figure 6:**
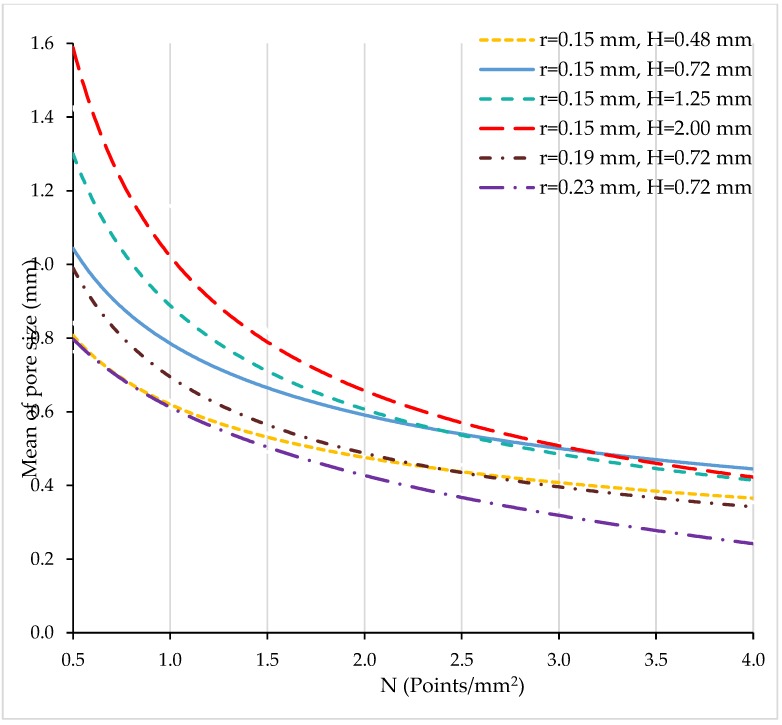
Mean of pore size (mm) vs. N (points/mm^2^).

In Figure 6, curves for the mean of the pore size are presented vs. N for different values of H and r.

The mean of the pore size decreases with the number of points with curves that exhibit asymptotic behaviour. Thus, the minimum achievable mean of the pore size ranges between 0.3 and 0.55 for the different conditions.

**Figure 7:**
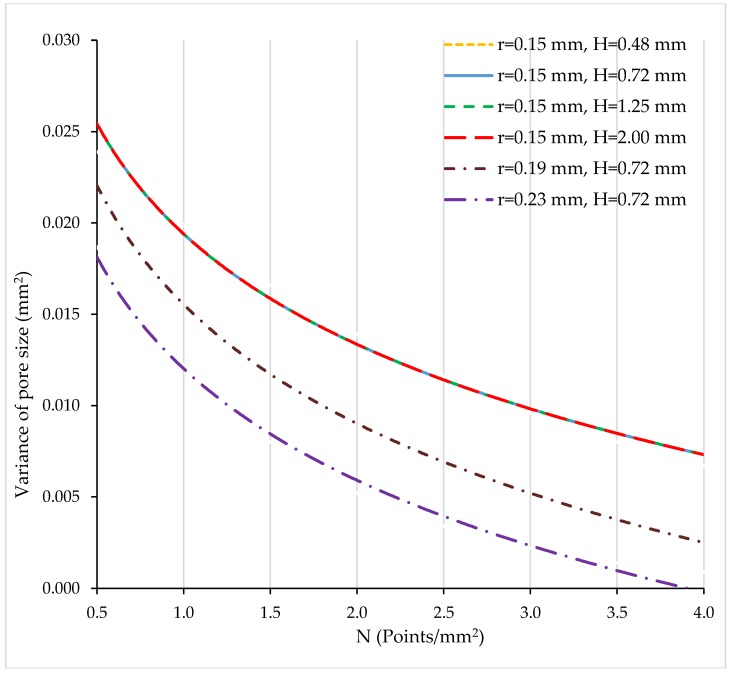
Variance of pore size (mm) vs. N (points/mm^2^).

Figure 7 shows curves for the variance of the pore size (mm^2^) vs. N (points/mm^2^).

The higher the number of points per unit area, the lower the variance of the pore size. The same variance values were calculated for all structures having r = 0.15 mm (the continuous curve is superimposed with the dashed ones). As the radius increases, the variance decreases. For r = 0.23 (dashed-dotted line in purple) and high N values, the variance values close to zero were obtained.

### 3.3. Experimental Tests

**Figure 8:**
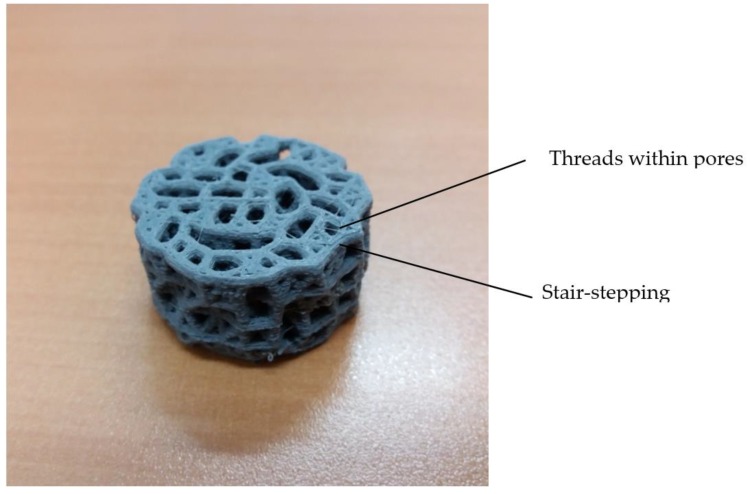
Printed porous structure (rescaling of the designed scaffold by scaling factor of five).

A picture of the printed sample, which is a rescaling of the designed scaffold by a scaling factor of five, is presented in Figure 8. It corresponds to the optimal solution of multiobjective optimization, with N = 3.65 points/mm^2^, r = 0.15 mm, and H = 0.72 mm.

Some defects of the printed structure are observed. Small threads within pores are caused by the fact that retraction or recoil movement of the filament to prevent dipping when it is not printing did not work properly. In addition, surface finish shows stair-stepping defect due to layer-by-layer construction, which is more which is more patent the higher the layer height used. The printing process will be improved in further works.

**Figure 9:**
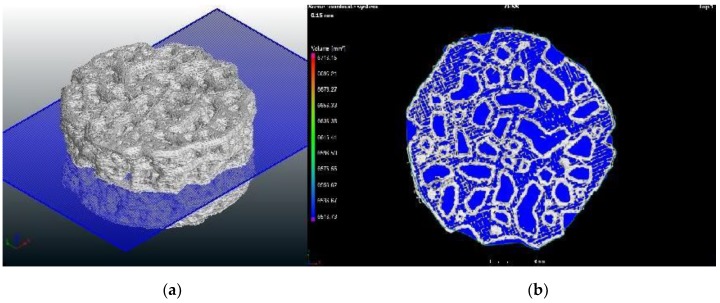
Cross-section of specimen 1: (**a**) 3D view, and (**b**) 2D view.

As an example, Figure 9a depicts the measured geometry of specimen 1 with a cross-section at the half height of the disk. Figure 9b corresponds to the geometry of the cross-section of specimen 1. Both were obtained by means of X-ray tomography.

Both quite round pores and more elongated pores are observed, with a larger pore group and a smaller pore group. This fact could be useful when both nutrient transport and osseointegration are required [35]. The length of the largest elongated pore of rescaled printed specimen 1 is close to 5 mm (which corresponds to 1 mm in the designed specimen), which is a too high a value, and needs to be reduced in further works. However, the width of the same pore of the rescaled printed specimen is about 1.5 mm (0.3 mm in the designed specimen), which lies within the selected interval for the pore size.

Measured porosity, measured mean of the pore size, and measured variance of the three specimens are presented in Table 6.

The average value of the measured porosity for the three specimens was 0.594, showing good agreement with the result from multiobjective optimization, 0.537. The average value of the mean of the pore size was 0.372 mm, which is lower than the result from optimization, 0.434 mm. However, it is close to the centre of the considered interval, 0.300 mm. Variance ranges from 0.035 mm^2^ to 0.067 mm^2^, which is higher than target value of 0.010 mm^2^. Nevertheless, this result could be desirable, since some variance of the pore size is required when both osseointegration and nutrient transport are required [35]. Thus, printed PLA structures comply with the requirements for porosity and the mean of the pore size in trabecular structures.

## 4. Discussion

Unlike other methods for defining porous structures, which are based on truss structures, for example with hierarchical scaffold design [11,12] or with topology optimization [16,17], in the present paper a new method is presented that allows obtaining irregular porous structures from random location of columns, which leave voids of different sizes among them. The structure is defined with parallel planes having several points on each plane, and lines that join points from different planes or from the same plane. This theoretical structure, which would be infinite, must be bounded in a geometric figure, in this case a disk, although it could be applied to other types of figures such as prisms, cubes, cylinders, etc. Afterwards, the lines are converted into columns of a certain radius with help of the marching cubes algorithm [29]. Thus, the geometrical model is converted into a porous structure. The planes can be designated as layers and the lines as columns. From the correct selection of values for the independent variables of the system, it is possible to obtain structures with different porosity and pore size. In this case, three variables were chosen, namely the distance between parallel layers H, the number of points per area unit that generate columns on each layer, N, and the radius of columns, r. In order to reduce number of variables involved in the problem, dimensional analysis is applied [30]. In this case, radius of columns was used as the scale variable and two dimensionless variables were obtained: H/r and N·r^2^.

Once the model depends on n-k dimensional variables (in this case n−k = 3 − 1 = 2 variables), it is possible to apply design of experiments in order to determine the influence of variables on selected responses. A full factorial design was selected, with three factors and three levels, with a total amount of 27 runs. In this work, responses were porosity, the mean of the pore size, and the variance of the pore size. Values for responses were calculated from simulated structures for each combination of variables. From results of design of experiments, by means of multiobjective optimization it is possible to select certain values for variables in order to achieve the required porosity and pore size values. Four different cases were taken into account, depending on importance given to each response. In this study, the desirability function method was employed, although other optimization methods are possible, either generating methods (no-preference, a posteriori, ...) or preference-based methods (a priori, interative, ...) [38].

In the present work, main objective of optimization is to assure fixation of the structure by means of osseointegration. For this reason, target values were defined for both the porosity and the mean of the pore size according to the bibliography, while the mean of the pore size is to be minimized so that similar pores are obtained. However, in the future the methodology can be applied to other objectives, for example, the strength of the structure and/or nutrient transport. As a general trend, the lower the porosity, the higher the mechanical strength of a structure [39]. In case nutrient transport is considered as a goal, then a certain value for the variance of the pore size is recommended. Smaller pores are required with high surface/volume ratio in order to favour cell attachment before osseintegration and larger pores are needed to help nutrient transport [35].

In order to test if it was possible to print designed structures, three specimens were printed in PLA by means of FDM technology. Measured porosity of 0.594 was similar to the computationally-defined value of 0.537. Measured mean pore size of 0.372 mm was lower than the simulated value of 0.434 mm. Thus, the measured results for porosity and pore size are quite similar to the calculated ones, and agree with the requirements, although unlike the designed structure, which has round pores, in printed samples both round and elongated pores were observed. The experimental average value of the variance of the pore size was higher than the calculated one, and this will be addressed in further works.

Further research is required in order to improve the printing process of the scaffolds. Future trends include using FDM techniques with a lower nozzle diameter (below 0.1 mm) in order to reduce the height of steps or controlling the flow in order to avoid filaments within the pores [40]. Other additive manufacturing technologies, such as stereolithography (STL), having higher dimensional accuracy than FDM, can also be employed [41].

## 5. Conclusions

In the present paper a new method for designing porous structures is presented. First, a geometric model is defined that is based on parallel layers with several columns of a certain radius that join them. The model can be applied to a certain shape, for example a disk. Then, by means of dimensional analysis, number of variables is reduced. In this case, two dimensionless variables are used: H/r and N·r^2^. Next, design of experiments allows obtaining mathematical models that relate process variables to responses, in this case the porosity (dimensionless), the mean of the pore size (mm) and the variance of the pore size (mm^2^) of the structures. Then, multiobjective optimization provides the values of the variables that allow obtaining required values for the responses.

If the model is applied to a trabecular structure, in order to obtain target porosity of 0.625, target mean of pore size of 0.300 mm and low variance of pore size, recommended values were found for model variables: lowest radius of columns r = 0.15 mm and a medium value for height between parallel surfaces of H = 0.72. The number of points ranges from N = 3.20 to 3.90 points/mm^2^ depending on the importance given to each response. For example, if the three responses have the same importance, N = 3.65 points/mm^2^ are recommended. Higher number of points is recommended when mean or variance of pore size is more important than the rest of the variables. On the contrary, lower number of points is recommended when porosity is more important than the rest of the variables. The porosity and the mean of pore size obtained in experimental tests printed in polylactic acid (PLA), 0.594 and 0.372 mm respectively, were in good agreement with the simulated porosity and mean of pore size of 0.537 and 0.434 mm, respectively. Experimental variance of pore size is higher than the calculated one.

For fixed values of the distance between layers and of the radius of columns, the greater the number of points on layers, the lower the calculated porosity and the mean of pore size. A larger number of columns means there is less room for pores, leading to lower porosity, as well as shorter distance among columns, with lower pore size. The variance of pore size also decreases with number of points per surface unit. By varying the distance between layers, the radius of the columns and the number of columns per unit area, structures with different porosity and pore size can be achieved. The higher distance between layers is the higher porosity and mean of pore size. The higher the radius of columns is the lower porosity and mean of pore size. Thus, the model will be very useful in defining the required values for geometric variables, which will allow a certain degree of porosity and pore size in porous structures to be obtained.

The main limitation of the methodology is that it only takes into account the porosity of the structures that is required to achieve osseointegration, but not their mechanical properties (elastic modulus, compressive strength) or their ability to transport nutrients (permeability, diffusivity). Another limitation is that the structures are difficult to print with FDM technologies because of the low diameter of columns which is only slightly higher than nozzle diameter employed. For this reason, in the present work geometry was rescaled by a scaling factor of five prior to printing. Surface finish presents the stair-stepping defect due to layer-by-layer deposition.

In future work, other requirements for structures, related to either mechanical strength or mass transport, will be addressed. In addition, improvement of the FDM printing process is required in order to obtain more accurate and smooth parts. For example, nozzle diameter can be reduced and nozzle retraction can be controlled in order to minimise the stair-stepping effect and the presence of threads within pores respectively.

## Figures and Tables

**Table 1:** Levels of the factorial design.

	Level 1	Level 2	Level 3
H/r	3.2	4.0	4.8
N·r^2^	0.054	0.072	0.090

**Table 2:** Selected values of parameters for optimization.

Response	Type of Desirability Function	Lower Value L	Target Value T	Upper Value U	b
Porosity	Of target value	0.500	0.625	0.750	1
Mean (mm)	Of target value	0.100	0.300	0.500	1
Variance (mm^2^)	Minimization	0.000	*	0.010	1

* means that a certain parameter was not used.

**Table 3:** Intervals for variables and m-values used for optimization.

	Interval	m
H (mm)	[0.48, 2.00]	300
N (points/mm^2^)	[0.30, 4.00]	740
r (mm)	[0.15, 0.41]	200

**Table 4:** Results of the porosity, the mean of the pore size, and the variance of the pore size.

Nr	H/r	N·r^2^	Porosity	Mean (mm)	Variance (mm^2^)
1	−1	−1	0.574	0.524	0.009
2	−1	0	0.491	0.439	0.009
3	−1	1	0.409	0.359	0.006
4	0	−1	0.599	0.549	0.012
5	0	0	0.537	0.450	0.011
6	0	1	0.472	0.378	0.005
7	1	−1	0.645	0.578	0.015
8	1	0	0.563	0.466	0.007
9	1	1	0.500	0.414	0.008
10	−1	−1	0.587	0.559	0.022
11	−1	0	0.473	0.414	0.008
12	−1	1	0.436	0.369	0.007
13	0	−1	0.635	0.547	0.008
14	0	0	0.552	0.482	0.012
15	0	1	0.484	0.401	0.008
16	1	−1	0.644	0.575	0.010
17	1	0	0.582	0.491	0.012
18	1	1	0.521	0.411	0.006
19	−1	−1	0.549	0.525	0.016
20	−1	0	0.477	0.447	0.010
21	−1	1	0.430	0.397	0.008
22	0	−1	0.621	0.542	0.009
23	0	0	0.516	0.439	0.006
24	0	1	0.462	0.376	0.006
25	1	−1	0.641	0.575	0.015
26	1	0	0.571	0.476	0.008
27	1	1	0.502	0.401	0.009

**Table 5:** Optimization results.

	H (mm)	N (Points/mm^2^)	r (mm)	Porosity	Mean (mm)	Variance (mm^2^)	k	Desirability
Equivalent importance	0.72	3.65	0.15	0.537	0.434	0.008	$k_{porosity}=k_{mean}=k_{variance}=1$	0.283
Porosity more important	0.72	3.25	0.15	0.573	0.470	0.009	$k_{porosity}=5$ $k_{mean}=k_{variance}=1$	0.378
Mean more important	0.72	3.87	0.15	0.517	0.418	0.007	$k_{mean}=5$ $k_{porosity}=k_{variance}=1$	0.336
Variance more important	0.72	3.88	0.15	0.517	0.417	0.007	$k_{variance}=5$ $k_{porosity}=k_{mean}=1$	0.276

**Table 6:** Measured porosity, mean of the pore size, and the variance of the pore size of the printed specimens.

Specimen	Measured Porosity	Measured Mean of Pore Size (mm)	Measured Variance of Pore Size (mm^2^)
1	0.595	0.383	0.035
2	0.605	0.352	0.043
3	0.581	0.380	0.067
Average	0.594	0.372	0.048
